# Proposed method for evaluation and categorization of functional capacity of children, adolescents, and adults with cardiac diseases to bring them in existing social justice system by creating the cardiac disability criteria

**DOI:** 10.1007/s12055-019-00895-y

**Published:** 2020-01-24

**Authors:** Smita Mishra, Rajesh Sharma

**Affiliations:** 1 Department of Pediatric Cardiology, Manipal Hospital, Dwarka Sector 6, Delhi, India; 2 Paediatric Cardiac Surgery, Jaypee Hospital, Sector 128, Noida, UP India

**Keywords:** Cardiac disability, Comprehensive cardiac disability score (CCDS), Heart disease

## Abstract

**Introduction:**

Emerging epidemiological trends in India indicate the rising burden of cardiovascular diseases (CVDs) demanding a need of a social support system. Yet, the list of 21 benchmark disabilities notified by the Department of Empowerment of Persons with Disabilities, Ministry of Social Justice and Empowerment, Government of India, does not include CVDs under the newly enacted Rights of Persons with Disabilities (RPWD) Act, 2016. While the RPWD Act 2016 has acknowledged the dynamic nature of disabilities associated with congenital diseases like thalassemia, it has also provided an opportunity to bring in “cardiac disability” under its tenets. This would allow India to adopt strategies for the benefit of cardiac patients in accordance with policies adopted by developed countries such as the United States of America (USA), the United Kingdom of Great Britain (UK), and Canada. This document is to initiate a thought process of recruitment of cardiac patients in the social justice system.

**Aims and objectives:**

(1) To define cardiac disability, (2) to categorize cardiac diseases/defects (groups A–C) according to severity and need for interventions, (3) to identify operated and unoperated patients with normal functional capacity and their eligibility to avail normal opportunities similar to their peer groups, (4) to create a comprehensive cardiac disability scoring (CCDS) system for disability certification based on subjective and objective evaluation of functional capacity and the corresponding heart disease category group, and (5) to create a reference literature for the issues of education, employability, insurability, and vocational counseling based on this document.

**Methodology:**

The evolution of this manuscript has been discussed in view of relevant observations made by a team of cardiologists, cardiac surgeons, intensivists, pediatricians, social workers, etc.

**Conclusion:**

This manuscript suggests a CCDS system to lay down criteria for disability status for eligible patients suffering from cardiovascular diseases. It intends to offer a unique scientific tool to address the psychosocial and socio-economic bias against patients with heart diseases of heterogeneous nature.

“Resourcelessness of our patients makes them stoic and trusting and that increases our responsibility several folds.” Prof (Dr) SS Kothari [1].

## Introduction

The hemodynamic abnormalities in children, adolescents, and adults with cardiovascular disorders (CVD) are known to have a heterogeneous natural history and outcome [1–7]. Due to the increasing availability of advanced and refined cardiac procedures, there is a decline in overall cause-specific mortality rates for all ages by at least 71% [3]. About 40–60% survivors may have suboptimal functional capacity (FC) even after interventions, requiring lifelong expert consultation, medical support, or multiple re-interventions [4–11]. These requirements would then need further scrutinization for cost of care, resource utilization, and quality of life in terms of physical as well as the psychosocial stress [1–11]. Such financial burdens are also due to exorbitant costs of cardiac rehabilitation or work loss in the family. Lack of non-discriminatory provisions for social security, such as health or life insurance and prioritized full employment, complicate the condition [1, 2, 5, 6, 8]. There are several countries extending disability benefits to cardiac patients [12, 13]. On the other hand, despite emerging epidemiological trends indicating the rising burden of CVDs, cardiac diseases are not included in the list of 21 benchmark disabilities notified by the Department of Empowerment of Persons with Disabilities, Ministry of Social Justice and Empowerment, Government of India, under the newly enacted “RPWD Act, 2016 [2–8, 14–18].” The RPWD Act, 2016—a reincarnation of the “Persons with Disabilities (Equal Opportunities, Protection of Rights and Full Participation) Act, 1995”—is pragmatic and permissive in nature. It ascertains that disabilities may be “dynamic” in nature and may run a capricious course. Due to this paradigm shift in definition, 21 diseases (including congenital diseases like thalassemia) are on the notified disability list. The act enables India to participate in policies, plans, and programs in accordance with the United Nations Convention on the Rights of Persons with Disabilities (UNCRPD) (to which India is a signatory).

This manuscript provides a scientific tool to lay down criteria to segregate normal and abnormal functional capacity amongst the cardiac patients. It has been developed on behest of a group of pediatric cardiologists, pediatric cardiac surgeon, adult cardiac surgeon, intensivists, pediatricians, and social workers.

## Aims/objectives

1. To define cardiac disability.
2. To categorize cardiac diseases/defects (groups A–C) according to severity and need for interventions.
3. To identify operated and unoperated patients with normal functional capacity and their eligibility to avail normal opportunities similar to their peer groups.
4. To create a CCDS system for disability certification based on subjective and objective evaluation of functional capacity and the corresponding heart disease category group.
5. To create a reference literature for the issues of education, employability, insurability, and vocational counseling based on this document.

## Methodology: conceptualization of draft

Modern cardiac care is technology-driven and cost-consuming and requires lifelong medical attention and monitoring. The incentive to lay down a cardiac disability document sprung from the combined effect of many factors. Many instances as noted below can be recounted from day to day medico-social experience. These contradictions are rampant even today in the twenty-first century.

1. *Reluctance to timely referral of a patient for intervention*: Eisenmenger syndrome, a catastrophic complication of simple shunt lesions, is seen even in affluent families due to traditional bias against surgery amongst the pediatricians as well as the parents.
2. *Sub-optimal financial assistance for underprivileged cardiac patients*: Support from government schemes (e.g., Rastriya Bal Swasthya Karyakram [RBSK]) and non-government organizations (NGOs) is limited to simple heart diseases and there are no provisions for prostheses, pacemakers, and subsequent operations. RBSK does not support Fontan surgery, conduit replacements, and pacemaker implantations.
3. *Extended hospital stay after a major cardiac surgery and expensive aftercare*: It leads to loss of daily wages for caretakers, spiraling them into debt-entrapment and distress financing to meet the demands of costly aftercare even if cardiac surgery was fully financed.
4. *No medical care for cardiac patients operated with the help of the government or NGOs, presenting with non-cardiac emergencies like meningitis and cerebral malaria*: These patients are considered high risk by local physicians and get referred to tertiary care centers which they may not afford. Many of them succumb to non-cardiac ailments.
5. *Cardiovascular disability is not specified as disability*: Financial assistance from specified funds for children with disabilities is not available to needy cardiac patients.
6. *Apathy to psychological issues like depression in grown-up with congenital heart diseases (GUCH)*: Low self-esteem, incomplete education, and a fear of the future at the adolescent age contribute to mental health issues which are ignored completely by parents and physicians.
7. *The issue of life and health insurance*: In the absence of any guidelines to identify functional capacity of HD, discretion in allowing or disallowing a policy is in the hands of insurance companies and agents. The guidelines are required to frame effective health and life insurance policies.
8. *Denial to avail normal opportunities despite a normal functional capacity (FC)*: They are prohibited from participating in competitive sports by parents and physicians and get rejected in job and visa interviews, etc. Few of adults with CHD who were operated late were denied leave for surgery and were often needed to discontinue their jobs.
9. *Denial to administer benzathine penicillin for secondary prophylaxis of acute rheumatic fever (ARF)*: Many patients with a history of ARF never receive BPG and present with rheumatic heart disease (RHD) accompanied with severe left ventricular (LV) dysfunction due to denial to give BPG by many pediatricians or physicians.

The inferences from aforesaid points were that a holistic approach was needed to break the cultural barriers and to empower these patients appropriately for their future life by identifying their functional abilities. Also, many authors in various publications acknowledged the role of cultural barriers, financial constraints, underutilized prophylactic measures, deficient insurance facilities, inhomogeneous distribution of tertiary care centers across the country, and lack of long-term cardiac rehabilitation programs, in restraining the delivery of cardiac care in India [1, 2, 5–9]. Consequently, a group of professionals started working on this document in 2009 in order to get monetary support for cardiac patients within the ambit of Garibi Sahayata Nidhi, a scheme launched in 2008 by the Government of Madhya Pradesh to support underprivileged patients with disabilities. After the 2011 Disability census, a list of 19 diseases, including thalassemia and sickle cell anemia, was already forwarded to be enlisted in “Specified Disabilities” under the newly proposed bill. The Right of Persons with Disabilities Bill 2014 was introduced in the Parliament on 7 February 2014 and was passed on 14 December 2016 [18]. Efforts to propose a consensus document on cardiac disabilities to the government have not done yet.

### Literature search

The 36th Bethesda guidelines provide the eligibility criteria to customize sport activities according to the type of HD and interventions. [19]. This allowed us to understand the importance of assessment of FC in a patient with heart disease. Subsequently, a document titled: “Manual for Doctors to Evaluate Permanent Physical Impairment” was accessed on the internet [20]. The manual was a compilation of recommendations made by a group of experts during the “National Seminar on disability Evaluation and Dissemination” convened in collaboration with the Director General of Health Services (D.G.H.S.) and World Health Organization (W.H.O.) at All India Institute of Medical Science (A.I.I.M.S), New Delhi, in 1981. Page number 24 of this manual mentioned briefly the evaluation of physical impairment due to cardiopulmonary diseases based on the New York Heart Association (NYHA) criteria [20]. These incidences compelled us to make a determined effort to provide recourse to this vulnerable patient population. In the late 1990s, there was a global demand for a social security system with enhanced guidelines for cardiac patients. Dr. David S. Celermajer and Dr. John E. Deanfield, Cardiothoracic Unit, The Hospitals for Sick Children, Great Ormond Street, London, addressed this issue in a publication in 1993. They wrote “As the pediatric cardiac successes of the modern era reach adulthood they face problems of both, a medical and social nature. In the absence of appropriate guidelines some individual companies have set their own policies for young adults with congenital heart diseases. Unfortunately, many other companies have no policies framed on this subject, and in these situations/ circumstances, it is the patients who are inappropriately disadvantaged” [21].

The social security listing of cardiovascular disability (“Cardiovascular Disability: Updating the Social Security Listings”) by the Social Security Administration, United States of America, was accessed later, inspiriting us to take the matter further with the government [13]. As discussed below, this document conceptualizes the heart disease group category system and subjective/objective criteria to elucidate a CCDS based on existing literature [22–52].

### Disability assessment

- *Definition of a person with disability* (RPWD Act 2016): “a person with long term *physical, mental, intellectual or sensory impairment* hindering the person’s full, equal and effective participation in society [18].”
- *Definition of cardiac disability*: “a reduction of productivity to 40% less than expected, in accordance with age and skill due to reduced residual working capacity or need for frequent rest due to chest discomfort, syncope, dyspnea, fatigue, palpitation and cyanosis, leading to absence from work, frequent admission to the hospitals to manage the symptoms of hypoxia, heart failure (HF) and/or arrhythmia [13, 18, 41–44].”
- *Temporary cardiac disability*: “a cardiac-disability may be ‘temporary’ due to imminent improvement in functional capacity of the heart after the reasonable intervention or medical treatment [13, 18, 41–43].”
- *Permanent disability*: “the status of disability secondary to the cardiac cause when history, records, and clinical examination suggest that features of disability are there for 12 months or more and are unlikely to be resolved even with medical intervention [13, 18, 41–43].”
- *Reasonable treatment*: “the ‘treatment’ available at a location where it can be accessible to the person at a reasonable cost, can be expected to result in a substantial improvement in functional capacity, has a high success rate and carries a low risk to the person [13, 18, 41–43].”

### Step by step process of disability certification of the patients having heart diseases

#### Broad overview

- *Application for disability certification* received by the empowered committee from the patient or family after a cardiac diagnosis made by qualified physicians.
- *Exact diagnosis obtained* by a cardiologist/pediatric cardiologist after reviewing history, review of records, clinical examination/appropriate investigations (heart rate [HR], respiratory rate [RR], blood pressure [BP], X-ray chest, electrocardiogram [ECG], Holter test, treadmill test [TMT], stress echo/stress thallium, echocardiography, computerized tomography [CT], angiogram, magnetic resonance scan [MRI], positron emission tomography [PET] scan, cardiac biopsy, ambulatory monitoring of blood pressure, investigations to evaluate kidney, liver brain).
- *Heart diseases are categorized as the heart disease category group (HDC Gr) A, B, and C*. An entry score is assigned (Table 1).
- *Within each category, the HDs are subdivided into five subdivisions* (1–5).
- *Subjective assessment* of FC is done and a subjective score is assigned (Table 2).
- *Objective assessment* and objective scores are assigned (Table 3).
- *The comprehensive cardiac disability score* (CCDS; Table 4) is calculated by summing up scores assigned in Tables 1, 2, and 3.
- Disability status (DS)/disability certificate (DC)—D0/D1/D2 is assigned.
- Enlisted HDs in HDC groups A, B, and C are shown in Tables 5, 6, and 7.
- *Patients with co-existing illnesses*: In patients with co-existing defects, the disability certification must be extended for the more severe disease, as in a case of Down’s syndrome, mental retardation may be the major disability. Patients also must undergo the assessment of development issues and evaluation of other organs like renal abnormalities and visual or hearing impairments.

**Table 1:** Score allocation for heart disease categories groups (HDC Gr) of cardiac diseases (groups A, B, and C) [2, 9, 13, 20, 22–50]

Heart disease category groups (HDC Gr)	Details of categories of cardiac diseases as diagnosed by cardiologist			Diseases category entry score
	Severity of hemodynamic abnormalities	Necessity of intervention	Expected outcome	
HDC Gr A^a^	Not significant	No	Good	+ 5
HDC Gr B^b^	Significant	May need eventually	Variable	+ 10
HDC Gr C^c^	Significant	Yes (if suitable for intervention)	Variable	+ 20

^a^Ref. Table 5

^b^Ref. Table 6

^c^Ref. Table 7

**Table 2:** Cardiac disability score—subjective criteria score

CDS-subjective criteria for above 14 years of age [13, 40–47] (maximum score = 40)	
+ 0	No symptoms (NYHA I) (1) can undertake age-appropriate exercise for at least 30 min at a time; (2) is able to complete any physically active task at home or outside.
+ 10	Symptomatic (NYHAII) (1) mild dyspnea, fatigue, and chest pain only occasionally in physically demanding activities—walking to a larger working place without stopping to rest; or performing physically active tasks (e.g., climbing a flight of stairs) or heavier household activities (e.g., sweeping floor or mowing the lawn); (2) is able to perform most work-related tasks, other than tasks involving heavy manual labor (e.g., digging, carrying or moving heavy objects, concreting, bricklaying, laying pavers). (3) acyanotic.
+ 20	Symptomatic (NYHA II–III) (1) experiences symptoms, e.g., shortness of breath, fatigue, cardiac pain) when performing day to day activities around the home and community and, due to these symptoms; (2) is unable to walk far outside the home or has difficulty performing day to day household activities (e.g., changing the sheets on a bed or sweeping paths); (3) is able to do tasks like use public transport and walk around in market and can perform work sedentary or stationary nature. (4) acyanotic/ no need for home oxygen therapy
+ 30	Symptomatic (NYHA III–IV) (1) The person usually experiences symptoms (e.g., shortness of breath, fatigue, cardiac pain): when performing light physical activities, the person requires assistance to perform light day to day household activities; (2) feels difficulty in sustaining a clerical, sedentary, or stationary for a continuous shift of at least 3 h; ± systemic desaturation SPO2 < 95%. Needing home oxygen therapy at exertion (arterial pO2 ≤ 55 mmHg or SPO2 O2 ≤ 88%)
+ 40 and above	Symptomatic (NYHA IV). (1) usually experiences symptoms (e.g., shortness of breath, fatigue, cardiac pain) even at rest unable to move around inside the home without the assistance, ± SPO2 < 95%; (2) needs home oxygen therapy at rest (arterial pO2 ≤ 55 mmHg or SPO2 O2 ≤ 88%)
CDS-subjective criteria for below 14 years of age (maximum score = 40)	
+ 0	Asymptomatic (Ross CHF scale grade 1), SPO2 > 95%
+ 10	Symptomatic (Ross CHF scale grade 2). Tachypnea but no gross alteration in feeding time/no diaphoresis; SPO2 > 95%;
+ 20	Symptomatic (Ross CHF scale grades 2–3). Tachypnea with diaphoresis with altered feeding time in infants/weight gain affected, dyspnea on exertion in older children; 2. SPO2 > 95%
+ 30	Symptomatic (Ross CHF scale grade 3). Marked tachypnea and diaphoresis with feeding, prolonged feeding times and growth failure in infants/marked dyspnea on exertion in children; and/or SPO2 < 95% in infants and children
+ 40	Symptomatic (Ross CHF scale grade 4). Tachypnea, retractions, grunting, or diaphoresis at rest in infants/marked symptoms in older children at rest; and/or history of cyanotic spell in infants or squatting in older children, ± SPO2 at room air < 95%; further fall in SPO2 on walking (older children)

**Table 3:** Disability score—objective criteria (for patients both above or below 14 years of age)

Scores from CDS-objective criteria [12, 13, 41–52] (maximum score = 40)	
+ 10	Basal ECG or Holter monitoring (± abnormal imaging echo/CT/MRI/PET/cardiac cath) suggestive of significant ischemia, strain, severe ventricular hypertrophy or rhythm disorders, channelopathies, needing intervention; ECHO/CT/MRI (± ECG changes) suggestive of HD (structural, coronary or vascular or pericardial or tumor) associated with serious hemodynamic effects due to anatomical/functional abnormalities; pre/post-intervention HD presenting with functional abnormalities(+ regional wall motion abnormality, LV function < 40%, high PA pressure > mean 40 mmHg/PVRI > 6 woods unit, RV Dysfunction by existing criteria-TAPSE less than expected age-appropriate value; Fr. area shortening = < 30%), ± significant residual lesions or systemic hypertension
+ 10	6-min walk test < 400 m (≃abnormal exercise test, MET< 7, V2Max < 14 ml/kg/min/) ± abnormal stress test (TMT/echocardiogram/thallium test) in presence of HD with significant hemodynamic challenges (exercise, stress test must not be done in critical diseases)
+ 10	For evidence of clinical HF(systemic congestion-raised JVP/hepatomegaly, pedal edema or pulmonary congestion/crepts/pulmonary edema, basal tachycardia, bradycardia, or chronotropic incompetence* (abnormal chronotropic index (< 0.8 and > 1.3) - peak HR/resting HR)/(220 - age/resting HR) resulting into radiological HF (cardiomegaly, pulmonary edema, PAH/PVH); biochemical markers of HF (NT-proBNP > 1700 pg/mL or BNP > 140 pg/mL) or systemic hypoxia = Hct > 55% in presence of cyanosis
+ 10	Hospital admission records—documenting palliative procedure/cardiac syncope, E/O tachy- or bradyarrhythmia, chronotropic incompetence; presence of PPI, ICD/intervention/decongestive therapy/ionotropic support/requiring non-invasive ventilation/pulmonary vasodilators or therapy for systemic hypertension

*ECG* electrocardiogram, *CHF* congestive heart failure, *PAH* pulmonary hypertension, *PVH* pulmonary venous hypertension, *NT Pro BNP* N-terminal pro BNP, *BNP* B-type natriuretic peptide, *Hct* hematocrit, *Hb* hemoglobin, *HR* heart rate, *PPI* pacemaker implantation, *JVP* jugular venous pressure, *MET* metabolic equivalent of task, *V2Max* maximum rate of oxygen consumption measured during incremental exercise, *EF*, ejection fraction, *CT* computed tomography imaging, *MRI* magnetic resonance imaging, *PET* positron emission tomography

**Table 4:** Comprehensive cardiac disability score (based on scores contributed by Tables 1, 2, and 3)

Disability status-Global Disability Scale (% disability)	CDS-HDC group entry (maximum score = 20) Table-1	CDS-subjective criteria (max score = 40) Table-2	CDS-objective criteria criteria (max score = 40) Table-3	CCDS-maximum score = 100	Disability Certificate (DC)	Expected physical activity
No or mild (< 40%)					D0/D1 (DS ≤ 40)	No or some restriction
Moderate (40–70%)					D1 (DS ≥ 40–70)	Some restriction
Severe (> 70%)					D1^a^/D2 (> 70)	Symptom restricted physical activity

*CDS* cardiac disability score, *CDS-HDC-Gr* CDS-heart diseases category group

^a^A hemodynamically severe diseases which can be corrected or palliated so that functional capacity can be improved can be given a D1 certificated till intervention happens. Subsequently a review is recommended after 12 months to check about the restoration of functional capacity and type of disability certificate. A 2-year review is recommended in all the cases when a variable course of a cardiac disease (treated, untreated, or palliated) is expected. Global Disability Scale = mild < 40%; moderate 40–70%; severe > 70%

**Table 5:** CDS-HDC group A (group entry score = + 5) no or mild hemodynamic abnormalities with least chances of progression and intervention (expected CCDS < 40; D0/D1 certificate)

A1. HD-unoperated	
• Patent foramen ovale (PFO)/small fenestrations at fossa ovalis with or without small aneurysm (< 10 mm) with no chamber dilatation and normal pulmonary artery (PA) pressure, no history of (h/o) cryptogenic syncope • Bilateral superior vena cava (SVC), left SVC to coronary sinus (LSVC-CS) with or without innominate vein but with intact coronary sinus roof • Levo-atrial cardinal vein with normal left atrial (LA) pressure • Isolated inferior vena cava (IVC) interruption with azygos/hemi-azygos continuation	
• Small fossa ovalis atrial septal Defect (ASD) < 6 mm without chamber dilation or pulmonary hypertension • Partial anomalous pulmonary venous drainage (PAPVC)of single pulmonary vein: without significant chamber enlargement/pulmonary arterial hypertension (PAH)/pulmonary venous hypertension (PVH) • Ebstein’s anomaly grade I/II with mild or low moderate tricuspid regurgitation (TR) with mild chamber enlargement and no atrial shunt • Cleft mitral valve (MV) with no regurgitation • Congenital MV defect without significant gradient/regurgitation/PVH or PAH • Bicuspid aortic valve (AV): with no more than mild regurgitation and mean pressure gradient no more than 20 mmHg, no aortic root dilatation • Valvar pulmonary stenosis (PS) peak gradient < 40 mmHg with no right ventricle (RV) pressure overload • Ventricular septal defects (exception-doubly committed VSD): small causing no pulmonary arterial hypertension/chamber enlargement or aortic valve (AV) prolapsed or aortic regurgitation (AR) • Patent ductus arteriosus (PDA): tiny, not audible even to expert’s ears or incapable of producing complete continuous signal on continuous wave (CW) Doppler and not associated with additional shunt lesion or bicuspid aortic valve or coarctation of aorta with normal echocardiographic evaluation of heart • Flow acceleration at branch pulmonary arteries/ isthmus of aorta (gradient < 18 mmHg) without diastolic spill and RV/left ventricle (LV) volume-pressure (*v*/*p*) overload • Mild narrowing of isthmus, or flow acceleration-gradient < 20 mmHg, no diastolic spill, no upper limb hypertension, stationary gradient over 6 months of follow-up	
A2. HD-underwent corrective surgery/intervention	
• Acyanotic heart diseases underwent appropriate interventions and have no ventricular dysfunction, dilatation, hypertrophy, significant valvular regurgitation, obstruction, rhythm disturbance, no or mild pulmonary arterial or venous hypertension, systemic hypertension • Few cases of post-op cyanotic congenital heart disease (CHD) like tetralogy of Fallot (TOF) without trans-annular patch (TAP): who do not have residual lesions or ventricular dilatation, hypertrophy, dysfunction, pulmonary hypertension, or arrhythmia • Post pulmonary valvotomy: peak gradient no more than 30 mmHg/no significant RA/RV dilatation or RV dysfunction was noticed on follow-up • MV repair for cleft/prolapse: with no more than mild MR. Normalization of LV-M mode indices on follow-up • Total/partial anomalous pulmonary venous drainage: repair without residual PAH/PVH/arrhythmia • Post-op anomalous origin of left coronary artery from pulmonary artery (ALCAPA), if there no ventricular dysfunction or mitral regurgitation or ECG changes on rest or on exercise	
A3. HD of hemodynamic significance which improved by palliative intervention	
• Viral myocarditis or dilated cardiomyopathy due to causes like hypocalcaemia: recovered fully with normal ventricular function/ normal chamber size and no more than mild MR regurgitation • Cardiac tumor like rhabdomyoma regressed completely • Mild hypertrophic cardiomyopathy (HCM): the patients having no significant intra cardiac gradients. No history of syncope, arrhythmia, or systolic anterior motion of mitral leaflet or MR and no progression over the period of time • Muscular VSD closed completely or became hemodynamically insignificant post PA band ± PA de-banding with normal RV pressure • Lesions (ASD/VSD/PDA/AS/PS/MR/TR) is static over the repeated cardiac evaluation and have no ventricular dilatation or PAH • Rheumatic heart disease (RHD): 1. regurgitant lesion reverted to no or mild regurgitation and patient is on continuous secondary prophylaxis • Kawasaki disease (KD): has coronary dilatation which has reverted back to normal on follow-up period. Normal ECG (electrocardiogram) and TMT (treadmill test) • Pulmonary stenosis valvar, supravalvar, sub-valvar: gradient < 30 mmHg peak; not increasing over the period of time • Aortic stenosis (AS)—s/o valvar, sub-valvar or supravalvar: gradient < 20 mmHg mean; static lesions, over the period of the time and no ventricular hypertrophy or dysfunction • Mitral stenosis (MS)/supramitral ring (SMR): mean gradient < 5 mmHg and remains static, no evidence of PAH or pulmonary edema, right heart dilatation, patient is on regular secondary prophylaxis • Heterotaxy syndrome without cardiac malformations or arrhythmia (isomerism of left atrial appendage may not have significant heart defect)	
A4. Systemic arterial hypertension /pulmonary arterial hypertension or pulmonary venous hypertension ± hemodynamically insignificant pre- or post-operative structural heart disease	
• Pulmonary hypertension: primary PAH, attitude PAH, post-op residual PAH (mean PAP < 30 mmHg), no RVH or RV dilatation, normal IVC • Borderline or mild systemic hypertension-well controlled on lifestyle management or one or combination of drugs	
A 5. Dominant arrhythmia ± hemodynamically insignificant pre or post-operative structural heart disease	
• No recurrence after initial few episodes needing medication or cured by radio-frequency ablation • Evidence of (E/O) WPW (Wolff-Parkinson-White syndrome) syndrome on electrocardiogram (ECG) but no episodes of SVT (supraventricular arrhythmia)

**Table 6:** CDS-HDC group B: (group entry score = + 10) significant hemodynamic effect, high chances of progression and intervention; expected disability: moderate to severe (expected CCDS 40–70)

B1. HD-unoperated	
• Pre-tricuspid shunt-fossa ovalis ASD; SV (sinus venosus) ASD; primum ASD (± small VSD, mild MR); CS (coronary sinus) ASD with chamber dilation mild or moderate pulmonary hypertension/no h/o palpitation, no significant MR or TR or pulmonary venous stenosis, no associated heterotaxy syndrome. PFO with cryptogenic stroke • Partial anomalous pulmonary venous drainage of one or two veins: with some chamber enlargement but no or mild PAH/PVH • Acyanotic post-tricuspid shunts-ventricular septal defects: doubly committed VSD/perimembranous/muscular/inlet VSD mild to moderate pulmonary arterial hypertension (PAP less than 2/3rd systemic)/with some chamber enlargement mild aortic valve prolapse (doubly committed VSD), with or without trivial aortic regurgitation; patent ductus arteriosus: continuous murmur/capable of producing complete continuous signal on CW Doppler and associated with no significant additional shunt lesion or obstructive or regurgitant bicuspid aortic valve with normal or borderline enlargement of LA/LV and mild to moderate PAH • Coronary AV fistula(AVF), pulmonary AV fistula with mild cyanosis • Ebstein’s anomaly grade I/II with moderate TR with significant chamber enlargement/right to left shunting at atrial level • Corrected transposition (c-TGA), ± mild to moderate PS, no TR, no arrhythmia, no LV regression • Cleft mitral valve/congenital mitral valve defect with moderate gradient / mild to moderate regurgitation but no PVH or PAH • Bicuspid aortic valve: with mild to moderate regurgitation with chamber dilation (LVIDd (Left ventricular internal dimension-diastole) < 3.8 cm/m^2^; LVIDs (LVID-systole) < 2.6 cm/m^2^) and/or aortic stenosis with mean PG no more than 40 mmHg and no significant LVH in presence of normal ventricular function • Valvar pulmonary stenosis peak gradient less than 60 mmHg with no significant RV pressure overload • Posterior shelf at isthmus, ± hypoplastic arch, gradient ≤ 20 mmHg, but mild diastolic spill, mild upper limb hypertension, no LVH (left ventricular hypertrophy), normal EF (ejection fraction) • Branch pulmonary arteries stenosis with mild RV hypertension Lung perfusion scan mildly abnormal • Unoperated rheumatic/degenerative valvar diseases with mildly symptomatic patients • Post Kawasaki small aneurysm of coronary artery disease no cardiac dysfunction, regional wall motion abnormality, MR • Asymptomatic constrictive Pericarditis diagnosed on echo/CT (computed tomography imaging)/MRI/cath—mild JVP rise or hepatomegaly, no ascites • Hypertrophic cardiomyopathy with LVOT obstruction < 35 mmHg or not symptomatic in more than routine activity. • Dilated cardiomyopathy LVEF > 40% • Asymptomatic restrictive cardiomyopathy mild or moderately raised PA pressure ▪ Coronary heart disease in adults with no significant symptoms and amenable to intervention. Systemic hypertension controlled with multidrug therapy with or without end organ effects. ▪ Marfan’s syndrome or other familial thoracic aortic aneurysm syndromes (Loeys-Dietz syndrome [due to TGFBR1 and TGFBR2 mutations] and those related to mutations in SMAD3, TGFB2, and TGFB3), if aortic root is dilated but < 40 mm and no significant mitral tricuspid regurgitation (due to nature of disease, it cannot be kept in Table 5).	
B2. HD-underwent corrective surgery/intervention (Evaluation after 12 months)	
Post-surgery congenital/valvar/pericardial/coronary heart disease. Evaluation after 12 months’ revealed persistent mildly or moderately increased chamber size and mild to moderate MR/AR or moderate to severe TR/severe PR/mild to moderate residual gradient across the treated valve/mild or moderate PAH/mild pulmonary venous stenosis of > 2 veins; LVEF 40-50%	
• Pre- or post-tricuspid shunt lesions, coronary AV fistula (post-intervention)-with residual shunt >2 mm, ventricular dilatation (LV Z score >2) mean PAP >25<40 mmHg • TOF/DORV (double outlet right ventricle)/D-TGA (D-transposition of great vessel or ventricular arterial discordance)/C-TGA (corrected-transposition of great vessels or atrio-ventricular or ventricular arterial discordance). Anatomical repair-with mild or no RVOT (RV outflow tract) obstruction or LVOT (LV outflow tract)obstruction/mild or low moderate regurgitation/no ventricular dysfunction, regional wall motion abnormalities/aortic root dilatation (Z score >+ 2 or progressive dilatation), mild or no ECG abnormality; residual shunt lesions (> 2 mm causing ventricular dilatation or Pulmonary hypertension) • Coarctation of aorta: neonatal or infantile coarctation: with mild residual gradient and systemic hypertension, ventricular hypertrophy, or dysfunction; coarctation of late childhood: stenting with no or mild residual lesion and hypertension • Bicuspid aortic valve: post ballooning: mean gradient < 40mmHG, aortic regurgitation. On follow-up, no echocardiographic or functional deterioration or more than mild dilatation or mild LVH was noticed • Post-op anomalous origin of any of coronary artery from pulmonary artery (ALCAPA) underwent corrective procedure and has mild ventricular dysfunction or MR	
B3. HD of hemodynamic significance which improved by palliative intervention	
• Systemic to pulmonary artery shunt, Glenn shunt; Fontan/TCPC (total cavo-pulmonary connection) surgery, symptomatic in more than routine activity or having basal SPO2 > 80% • Shunt procedure or RV to PA conduit palliation ± with or without unifocalization of MAPCA (major aorto-pulmonary collateral artery)-as a final procedure for complex CHD with hypoplastic ± nonconfluent ± absent central pulmonary arteries • PA banding as a final procedure for complex CHD or multiple VSDs or VSD with tricuspid or mitral valve straddling or unbalanced AVSD (atrioventricular septal defect) with SPO2 > 85%, without AV regurgitation, cardiac dysfunction • Palliative arterial switch or Senning done for TGA + VSD or DORV + subpulmonary VSD	
B4. Systemic arterial hypertension/pulmonary arterial hypertension or pulmonary venous hypertension ± hemodynamically insignificant pre or post-operative structural heart disease	
• Primary Pulmonary hypertension or post-operative residual PAH: PA mean pressure < 40 mmHg. • Systemic hypertension with or without mild end organ defect on multidrug therapy. Normal LVEF.	
B5. Dominant arrhythmia ± hemodynamically insignificant pre- or post-operative structural heart disease	
(Controlled but not cured) • Bradycardia not needing pacemaker • Bradycardia with pacemaker • Primary tachyarrhythmia controlled on adequate medical therapy or amenable to RFA, long QTc with no episodes of ventricular tachycardia • Complete heart block with narrow QRS complex escape rhythm and HR > 70/min, without chamber dilatation and cardiac dysfunction • CHB with pacemaker • Long QT syndrome (LQTS), Brugada syndrome (BrS), early repolarization syndrome, short QT syndrome, and possibly idiopathic VF Catecholaminergic polymorphic ventricular tachycardia (CPVT)

**Table 7:** CDS-HDC group C: (group entry score = + 20) significant hemodynamic abnormalities; intervention required if it is possible (expected CCDS > 70)

C 1. HD-unoperated	
• Pre-tricuspid shunt (amenable to intervention)-atrial septal defect/PAPVC (partial anomalous pulmonary venous connection) ± RV dysfunction, or AV valve regurgitation or rhythm dysfunction or moderate to severe pulmonary hypertension • Post-tricuspid shunt. With controlled or uncontrolled CHF-ventricular septal defect, AP window, patent ductus arteriosus, large coronary AV fistula, complete atrioventricular septal defect (AVSD) with balanced/unbalanced ventricles ± AV valve regurgitation ± LVOT or RVOT obstruction • Shunt lesion with SPO2 < 95% saturation on room air (Eisenmenger syndrome) • Obstructive lesions (sub-valvar, valvar, supravalvar-AS/PS) and vascular (pulmonary artery stenosis/coarctation of aorta) defect with pressure overload of respective ventricular chamber, with or without ventricular dysfunction (amenable to cath/surgical intervention) or MS, supravalvar MS with dilatation of left atrium, pulmonary venous congestion, RVH, TR, PAH; TS with RA dilatation, systemic venous congestion • Regurgitant lesions: mitral, aortic, tricuspid regurgitation (needing intervention according to the existing guidelines), pulmonary regurgitation with right heart dilatation • Large coronary AV fistula needing neonatal or infantile intervention • Cyanotic congenital heart disease (± arch anomaly)-Amenable for surgical intervention-tetralogy of Fallot (TOF); DORV + VSD ± RVOTO(RVOT obstruction)/LVOTO(LVOT obstruction); transposition of great arteries ± VSD, ± PDA ± LVOTO; corrected transposition with VSD ± PS with or without systemic severe TR; AVSDPS or PAH; AVSD-unbalanced ventricles; truncus arteriosus (TrA) with or without truncal valve stenosis or regurgitation; TrA with arch interruption; tricuspid Atresia/VSD+/-PS; Single ventricle VSD ± PS; TAPVC ± obstruction; heterotaxy syndrome with hemodynamically significant cardiac malformations; hypoplastic left heart syndrome and all complex congenital heart diseases with SPO2 < 90% ± cyanotic spell ± congestive heart failure • Cyanotic CHD with PAH/PS with small or absent PA’s large MAPCA-unsuitable for surgical or cath intervention-Eisenmenger Syndrome with Cyanotic CHD (increased blood flow-DORV or TGA + VSD without PS, single ventricle/tricuspid atresia without PS, TAPVC, truncus arteriosus) • Complex left-sided obstructive lesions like congenital mitral stenosis • Pulmonary AV fistula-significant systemic desaturation, sometimes involving significant part of lung and show poor outcome after intervention • Complex anatomy (unsuitable anatomy) mitral stenosis/regurgitation with or without ventricular hypoplasia, Shone complex in small children; veno-occlusive disease of pulmonary veins • Systemic very large-cranial AV fistula needing early intervention • Ebstein’s anomaly with severe TR, unguarded TV ± PFO/ASD, severely dilated RA + right to left shunt at PFO; pulmonary atresia with or without VSD; single ventricle PS/PAH • Cardiac tumors ± arrhythmia or diastolic dysfunction • Uhl’s anomaly of RV/RA appendage aneurysm • Rheumatic/degenerative valvar diseases with persistent congestive heart failure • Post Kawasaki significant aneurysmal or stenotic coronary artery disease leading to moderate to severe cardiac dysfunction, regional wall motion abnormality, MR • Premature coronary artery disease in children and adolescent (vasculitis syndromes, metabolic syndrome hypercholesteremia) with persistent LV dysfunction or suboptimal result of revascularization surgery or intervention are expected • Coronary heart disease in adults with significant angina, dyspnea on exertion, or heart failure • Symptomatic constrictive pericarditis more than NYHA class II symptoms • Hypertrophic (obstructive) cardiomyopathy (HCM/HOCM) with LVOT obstruction > 35 mmHg or symptomatic more than routine activity (particularly if there is history of previous cardiac arrest, syncope, ventricular arrhythmias, family history of sudden death, extreme LV hypertrophy, and a blunted blood pressure response to exercise • Dilated cardiomyopathy (DCMP) LVEF < 40%, chamber dilatation, partially controlled or uncontrolled CHF ± MR • Restrictive cardiomyopathy if symptoms are happening in routine activity and there is moderate to severe pulmonary hypertension	
C2. HD-underwent corrective surgery/intervention (Evaluation after 12 months of intervention)	
• Post-op patients with hemodynamically significant residual lesions (valvar stenosis or regurgitation), ventricular dysfunction, systemic desaturation (may or may not be amenable to surgery) • Operated cyanotic and acyanotic CHD with non-resolving pulmonary hypertension requiring medical management • Post coarctation/aortic repair or intervention (ballooning, stenting or vascular graft) ± residual gradient with significant systemic hypertension • Post CABG or coronary stenting with no recovery of ventricular dysfunction or presence of ventricular arrhythmia • Post heart or heart and lung transplant	
C 3. HD of hemodynamic significance which improved by palliative intervention	
• Systemic to pulmonary artery shunt, Glenn shunt, with significant systemic desaturation • Fontan/TCPC with complications like liver cirrhosis, plastic bronchitis, protein-losing enteropathy (PLE), systemic desaturation (SPO2 < 80%) • Primary pulmonary hypertension with or without palliative procedure like ASD creation or Pott’s shunt • PA banding as a final procedure for complex CHD or multiple VSDs or VSD with tricuspid or mitral valve straddling or unbalanced AVSD, presenting late with symptoms, not amenable to biventricular repair, systemic desaturation (SPO2 < 80%) or persistent CHF • Shunt procedure or RV to PA conduit palliation ± with or without unifocalization of MAPCA (major aortopulmonary collateral artery)-as a final procedure for complex CHD with hypoplastic ± nonconfluent ± absent central pulmonary arteries • Palliative arterial switch or Senning operation done for TGA + VSD or DORV + subpulmonary VSD	
C4. Systemic arterial hypertension /pulmonary arterial hypertension or pulmonary venous hypertension ± hemodynamically insignificant pre or post-operative structural heart disease	
• Primary pulmonary hypertension with symptoms or post-op residual PAH (a resting mean pulmonary artery pressure (mPAP) of ≥ 30 mmHg and a normal pulmonary capillary wedge pressure (PCWP) of ≤ 15 mmHg, PVR > 240 dyn × s × cm-5). • PPH with severe RV dysfunction. Severe systemic desaturation in presence of natural or therapeutic shunt. • Systemic hypertension with secondary organ dysfunction, cardiac dysfunction	
C 5. Dominant arrhythmia ± hemodynamically insignificant pre or post-operative structural heart disease	
• Primary channelopathies, tachy-/bradyarrhythmias with history of life-threatening arrhythmia or with ICD • Arrhythmogenic right ventricular dysplasia-with or without history of syncope and arrhythmia, severe RV dysfunction • Non-resolving post-op arrhythmia in presence of ventricular dysfunction, hypertrophy, dilatation, pulmonary venous or arterial hypertension • Any CHD/VHD or coronary heart disease with ICD implantation • Any treated or untreated CHD/VHD with rhythm disturbances requiring EP study, RFA which is expected to have sub-optimal outcome • Primary rhythm disturbance with poor outcome of EP procedures • Intractable primary tachyarrhythmia, long QTc with episodes of ventricular tachycardia • Complete heart block	

### Summary (step by step process of disability certification of the patients having heart diseases)

- Heart diseases categories—HDC groups A, B, and C and their entry score (Table 1)
- Subjective criteria score (Table 2)
- Objective criteria score (Table 3)
- Comprehensive cardiac disability score—CCDS (Table 4)
- Detailed list of heart diseases categorized into HDC groups A, B, and C (Tables 5, 6, and 7)
- HDC group subdivision: 1–5 (*see below*)

### Heart diseases (HD) and heart diseases categories (HDC)—groups A, B, and C [2, 3, 5–7, 9, 13, 22–54]

HD in a patient may be isolated or a combination of congenital heart disease (CHD); valvular heart disease (VHD); myocardial, pericardial, coronary arterial diseases (CAD); cardiac tumors; rhythm disorders; and pulmonary arterial hypertension (PAH)/systemic hypertension (SAH). Details of HDC group’s entry scores are given in Table 1.

HDC Gr A, B, and C are formed on three common variables:

1. Severity of hemodynamic abnormalities
2. Necessity for intervention
3. Expected outcome

#### Heart diseases category group A—expected CCDS < 40 (Table 1)

Patients in the HDC Gr A are expected to have normal or near normal hemodynamics and FC and they are assigned a group entry score of + 5. According to the clinical evidence and investigations they fall into the following groups:

1. Asymptomatic non-progressive minor heart diseases, not needing intervention and have good long-term outcome.
2. Post-intervention HD with the restored FC; the patients are not expected to deteriorate in the future under usual circumstances and will not need intervention and have good long-term intervention.

A detailed list of HDs included in the HDC group A is given in Table 5.

#### Heart disease category group B—expected CCDS 40–70 (Table 1)

Patients in HDC Gr B are assigned a group entry score of + 10. They have or expected to have moderate to severe hemodynamic abnormality and abnormal FC. Clinical analysis and investigations may suggest one of the following:

1. A HD needs no intervention for now, but may have a chance of progressive deterioration hemodynamically affecting FC adversely and may need intervention in future, e.g., moderate aortic stenosis (AS).
2. The HD with significantly abnormal hemodynamics and moderately affected FC but can be expected to restore it after intervention. (e.g., large atrial septal defect [ASD]/moderate pulmonary stenosis [PS])
3. The post-intervention-HDs with restored FC have clinical evidence to suggest possible deterioration later in life due to (a) lack of age-appropriate growth disproportionate growth of repaired part (e.g., supravalvar aortic stenosis or aortic root dilatation after the arterial switch operation), presence of progressive ventricular (right ventricle [RV] or left ventricle [LV]) enlargement or hypertrophy (e.g., post tetralogy of Fallot [TOF] repair, reduced ejection fraction (e.g., post coronary artery re-implantation for anomalous origin of coronary artery from pulmonary artery [ALCAPA]), rhythm issues (e.g., atrial flutter post atrial septal defect [ASD] repair), or residual PAH (e.g., ventricular septal defect [VSD] closure) and (b) future complications are expected with valve or conduit placement surgery, e.g., post mitral valve replacement (MVR), conduit implantation for tetralogy of Fallot (TOF), coronary stenting, and Fontan surgery.
4. The HD underwent a palliative procedure to restore FC temporarily, waiting for complete anatomical (e.g., pulmonary artery banding [PAB] for multiple VSD) or physiological repair (e.g., post Glenn surgery).

A detailed list of HDs included in the HDC group A is given in Table 6.

#### Heart diseases category group C—expected CCDS > 70 (Table 1)

Patients in HDC Gr C are assigned a group entry score of + 20. They have progressive HD, expected to have moderate to severe hemodynamic abnormality, and have reduced FC.

The clinical and investigational analysis suggests significantly altered cardiac output, systemic hypoxia, ventricular dysfunction, arrhythmia, and significant PAH or SAH and suboptimal outcome is expected, even if intervention is done, leading to persistent severe restriction of FC. Many of them may need continuous medical support to perform ordinary activities.

A detailed list of HDs included in the HDC group A is given in Table 7.

#### Subdivision of heart disease category groups

HDC Gr A, B, and C are further subdivided into five subdivisions. The subdivisions do not influence disability score.

1. Heart diseases (HD)—unoperated
2. Heart disease (HD) with corrective surgery or intervention
3. Heart disease (HD) of hemodynamic significance which improved by palliative intervention
4. Systemic arterial hypertension (SAH)/pulmonary arterial hypertension (PAH) or pulmonary venous hypertension (PVH) ± hemodynamically insignificant pre- or post-operative structural heart disease
5. Dominant arrhythmia ± hemodynamically insignificant pre- or post-operative structural heart disease

### Cardiac disability score—subjective criteria (Table 2) [13, 18–20, 40–47]

Table 2 contains variables of CDS subjective criteria scores. Maximum contributory score allowed for this table is 40. The scores are based on NYHA functional classification for patients above 14 years of age [41]. For pediatric age group (≤ 14 years of age) the Ross Modified Classification for Heart Failure has been adapted [42].

### Cardiac disability score—objective criteria [12, 13, 41–49]

Table 3 contains variables of CDS subjective criteria scores. Maximum contributory score allowed in this table is 40. The criteria are based on standard objective cardiac assessments of the patient and hospital records.

### Comprehensive cardiac disability score disability scale and disability certificate (Table 4)

CCDS is calculated by summing up HDC group entry scores (Table 1), subjective criteria scores (Table 2), and objective criteria scores (Table 3). Maximum CCDS can be 100 which can be rated on a disability scale (equivalent to global disability score) for percentage disability (Table 1).

### Disability certificates (Table 4)

**Fig. 1:**
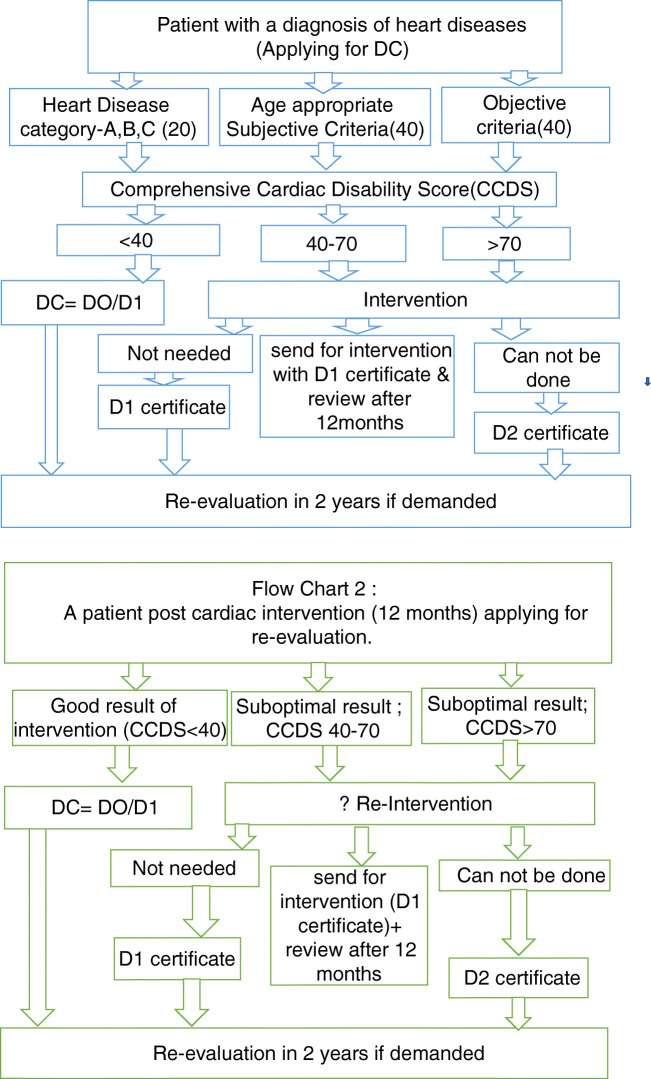
Cardiac disability certification flow charts 1 and 2

“Disability status” and type of disability certificates (DC) will be decided by the CCDS. As discussed before, the cardiac disabilities are dynamic by nature, thus needing revision as and when required. Therefore, disability status needs to be re-evaluated every 2 years, allowing the disability status to be upgraded or downgraded for the given patient. An exception can be made for patients with cardiac disabilities like Eisenmenger syndrome with no chance of recovery, by extending the duration for renewal of DC. The process of certification is summarized in flow charts 1 and 2 (Fig. 1).

#### Certificate D0

Patients with CCDS < 40 are expected to have less than 40% impairment of FC on the Global Disability Scale. They are accorded with certificate D0 which predicts none or mild disability. They must be considered comparable to peer group and may be awarded a fitness certificate if they demand.

#### Certificate D1 (temporary disability certificate)

- Patients with CCDS 40–70 are expected to have 40–70% impairment of functional capacity of the Global Disability Scale.
- Patients with CCDS > 70 (> 70% impairment of FC on the Global Disability Scale) having a HD which can be treated with intervention to restore FC must be accorded a D1 certificate and must be sent for intervention.
- Patients with CCDS < 40, but with a lesion which may deteriorate over the period of time due to the nature of disease or due to the underlying disease process (e.g., mitral valve [MV] prolapse without mitral regurgitation [MR] in a patient with Marfan syndrome).

#### Certificate D2 (permanent disability certificate)

If a patient with HD of hemodynamic significance is unsuitable for any successful intervention to improve the functional capacity or suboptimal results were achieved by intervention and no further intervention is possible, D2 certificate can be awarded.

- Patients with CCDS > 70 (> 70% impairment of functional capacity on Global Disability Scale).
- Patients with CCDS 40–70 (40–70% impairment of functional capacity on Global Disability Scale) who are unsuitable for interventions and are expected to be dependent on lifelong drugs, devices, or any other therapy to maintain their routine can be considered for D2 certificate. Examples are post-cardiac transplant patients, patients with implantable cardioverter-defibrillator, and patients with Eisenmenger syndrome.

#### Prefixing the disease category along with the disability certificate

In addition, the patient categorization of diseases (group A1–5, B1–5, C1–5) would add further specificity if it is prefixed to the disability certificate, e.g., A2D0 (HDC group A, subdivision 2, disability certificate D0) would imply a patient with a significant cardiac lesion who has been successfully operated and has no disability, while C5D1 (HDC group C, subdivision 5, disability certificate 1) would mean that it belongs to a patient with serious underlying heart disease with an ongoing rhythm issue but which may improve by the intervention (disability certificate D1).

Table 8 provides few more examples of the prefixing of respective HDC group and its subdivisions with appropriate disability certificates.

**Table 8:** Case scenarios: illustrations

Case scenario (disability certificate ( D-0,1,2) prefixed with HDC category -A,B,C and subdivisons 1-5)	Description: heart disease category group (HDC Gr); subdivision; comprehensive cardiac disability score (CCDS); expected disability certificate (DC)	Diagnosis
1. A 5-year-old child was certified as AIDO	HDC Gr A (no or mild hemodynamic abnormalities with least chances of progression and intervention) Subdivision: 1 (HD-unoperated) Expected CCDS: < 40 (< 40% 0n disability scale) Expected DC: D0 (predicts none or mild disability)	Restrictive perimembranous VSD
2. A 5-year-old child with certificate as B3D1	HDC Gr B (significant hemodynamic effect not needing intervention now but, high chances of progression, and intervention in future) Subdivision: 3 (HD with hemodynamic significance but improved by palliative intervention) Expected CCDS: 40–70 (40–70% on disability scale) Expected DC: D1 (it is possible to improve functional capacity significantly by doing an intervention)	Post Glenn shunt SPO2 = 85%
3. A 5-year-old patient as B4D1	HDC Gr B (significant hemodynamic effect, not needing intervention now but , high chances of progression and intervention in future) Subdivision: 4 (systemic arterial hypertension/pulmonary arterial hypertension or pulmonary venous hypertension ± hemodynamically insignificant pre or post-operative structural heart disease) Expected CCDS: 40–70 (40–70% on disability scale) Expected DC: D1 (it is possible to improve functional capacity significantly by doing an intervention)	Primary pulmonary hypertension (PA mean 35 mmHg)
4. A 5-years-old child certified as B1D1	HDC Gr B (significant hemodynamic effect, high chances of progression and intervention) Subdivision: 1(HD-unoperated) Expected CCDS: 40–70 (40–70% on disability scale) Expected DC: D1 (it is possible to improve functional capacity significantly by doing an intervention)	Large fossa ovalis ASD, no PAH/arrhythmia
5. A 45-year-old male patient as B3D1	HD Gr B (significant hemodynamic effect, not needing intervention now but high chances of progression and intervention in future) Subdivision: 3 (HD with hemodynamic significance but improved by palliative intervention) Expected CCDS: 40–70 (40–70% on disability scale) Expected DC: D1 (it is possible to improve functional capacity significantly by doing an intervention)	Post coronary artery stenting doing well. LVEF 40%

### Benefits for the patients grouped under the disability grading system

#### Prioritization of CVD in national health policies and funding

Disability status of cardiac patients will enable policy action to create a cost-effective population-based integrated health promotion programs and treatment strategies [18].

#### Disability benefit to individual patients

Patients with certificate D2 must be offered the same facilities as are provided under the “Rights of Persons with Disabilities Act, 2016 [18].”

#### Building future policies

1. *Health and life insurance* [4, 8, 13, 20, 52]: The group listing and disability certification (D0) help in identifying patients who are hemodynamically and functionally equivalent to the peer groups. They may have a legal right to procure a health or life insurance at a standard premium for the holders of D0 certificate while appropriately designed extra-premium can be offered to those who have D1 certificate.

2. *Guidelines for sports activities* [2, 53, 54]: Patients who have D0 certificates may participate in any sports activity (provided eligible under the screening guidelines for peer group). Those with the D1 certificate would need more stringent screening to be eligible for sports high in static and/or dynamic components. Many patients with the D2 certificate may be eligible for sports low on static and dynamic components.
3. This document can be used for counseling for other social aspects [2, 13, 54].

## Discussion and review of literature

### Prevalence of heart diseases

#### Congenital heart diseases

**Fig. 2:**
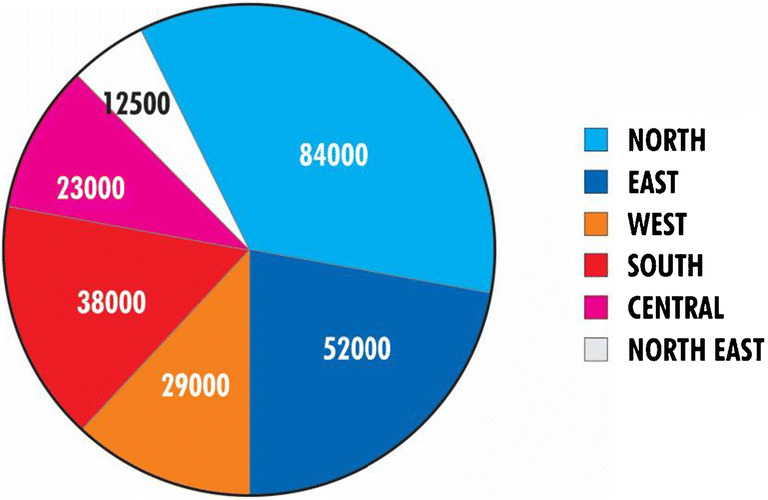
Regional distribution of infants born with CHD in India every year. Reproduced from [5]; reproduced with permission, Copyright© 1999–2019, Indian Pediatrics

In a recent single-institution study, the prevalence of neonatal heart disease was found to be 8.07/1000 live births while 44% of these babies had significant HD [2]. Table 9 shows the prevalence rate of CHD (0.3 to 9.2/1000 population) in older children, as reported in various published series [5]. The crude birth rate varies in various parts of India, and accordingly, the number of babies born with CHD shows a marked regional variation (Fig. 2) [5]. According to the status report on CHD in India (2018), approximately 27,000 patients (9700 infant and 1700 neonates) underwent interventions in 47 tertiary pediatric cardiac care centers in the years 2016–2017 [5]. Probability to access appropriate interventions for critical heart disease varied regionally and it was higher in the south (72%) and west (28%) and lower in the north (17%), central (7.6%), and northeast (0%) regions [5]. Only one fourth of needy infants born every year could access optimum cardiac care [5]. It is estimated that about 60,000 to 90,000 new cases of CHD are added every year to the existing pool of patients [5, 24]; 70–90% of them are expected to survive if they have access to appropriate modern health care [2–4]. Besides the CHDs, other heart diseases like CAD, rheumatic heart disease (RHD), cardiomyopathies (CMP), rhythm disorders, cardiac tumors, and pericardial diseases have a significant presence in India [1, 2, 5–7].

**Table 9:** Prevalence of congenital heart diseases in children

Studies	Age group (year)	Setting	Place of study	Total No.	Screening method	No. with CHD	Prevalence per 1000
Gupta et al. 1992	6-16	Community	Jammu	10,263	Clinical	8	0.8
Vashishtha et al. 1993	5-15	School	Agra	8449	Clinical	44	5.2
Thakur et al. 1995	5-16	School	Shimla	15,080	Clinical	30	2.25
Chadha et al. 2001	< 15	Community	Delhi	11,833	Clinical	50	4.2
Misra et al. 2009	4-18	School	Eastern Uttar Pradesh	118,212	Clinical Echo for suspected cases only	42	1.3
Kumari et al. 2013	5-16	School	Dist. Prakasam, Andhra Pradesh	4213	Clinical and echo in all	39	9.2
Saxena et al. 2013	5-15	School	Ballabgarh, Haryana	14,716	Clinical Clinical and echo	3577	2.37 5.23
Bhardwaj et al. 2016	All age/<18years	Community	Himachal Pradesh	1882/760, <18years	Clinical; echo in suspected cases	12 All/9.89<18years	6.31 (all) 12.95(<18 years)

Reproduced from [5]; reproduced with permission, Copyright© 1999–2019, Indian Pediatrics

#### Rheumatic heart disease (RHD): primordial prevention and secondary prophylaxis

**Fig. 3:**
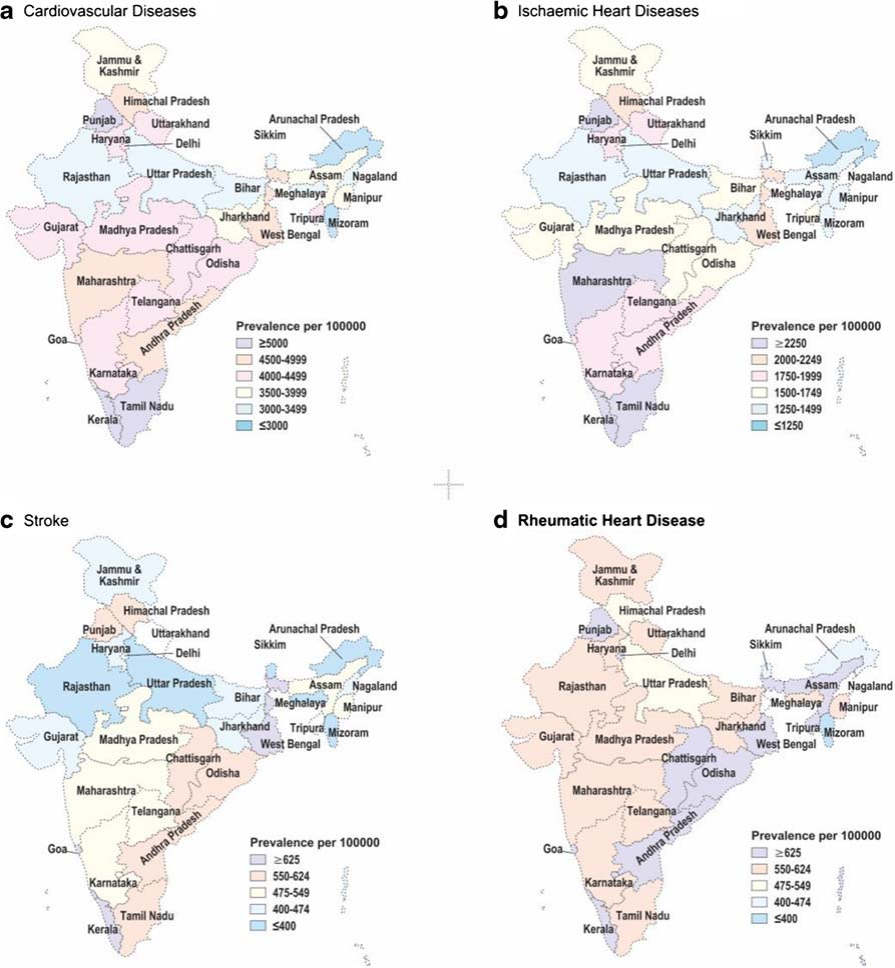
**a**–**d** Crude prevalence of cardiovascular diseases and major component causes in the states of India. Source: [15] (Fig. 2 reproduced under Creative Commons Attribution License [CCBY])

The estimated prevalence of RHD is less than 1/1000. Moreover, subclinical or silent carditis is expected to be 10 to 20 times higher than manifest RHD and has a potential to escape from secondary prophylaxis, if undetected [6]. A significant difference was reported on the prevalence of RHD in school children screened simultaneously by clinical examination (0.6/1000) and echocardiography (20/1000) [7]. The data from the Global Burden of Disease Study (GBDS) India 2016 revealed that the prevalence of RHD was 2.4 times the global average. Yet, the relative contribution of RHD to CVD-related percentage mortality (1.1%) and percentage DALYs (disability-adjusted life-year 0.8%) has declined (Table 10) [15]. Furthermore, RHD is now less prevalent in high economic transition level states (ETL; Fig. 3) [15].

**Table 10:** Percentage of total deaths and DALYs due to each cause under cardiovascular diseases by sex in India, 2016

	Percentage of total deaths (95% UI)			Percentage of total DALYs (95% UI)		
	Both sexes	Men	Women	Both sexes	Men	Women
Cardiovascular diseases	28.1% (26.5–29.1)	29.2% (27.5–30.3)	26.7% (23.8–28.3)	14.1% (12.9–15.3)	15.8% (14.5–17.1)	12.2% (10.9–13.4)
Ischemic heart disease	17.8% (16.8–18.5)	19.6% (18.5–20.4)	15.6% (13.9–16.6)	8.7% (7.9–9.5)	10.4% (9.5–11.3)	6.6% (5.9–7.4)
Stroke	7.1% (6.6–7.5)	6.9% (6.4–7.3)	7.3% (6.5–7.9)	3.5% (3.2–3.9)	3.6% (3.3–4.0)	3.4% (3.0–3.8)
Hypertensive heart disease	1.3% (1.1–1.5)	1.1% (0.9–1.4)	1.6% (1.2–1.9)	0.6% (0.5–0.7)	0.6% (0.5–0.7)	0.7% (0.5–0.8)
Rheumatic heart disease	1.1% (1.0–1.2)	0.8% (0.7–0.9)	1.5% (1.3–1.7)	0.8% (0.7–0.9)	0.7% (0.6–0.7)	1.0% (0.9–1.1)
Atrial fibrillation and flutter	0.21% (0.16–0.26)	0.17% (0.13–0.21)	0.25% (0.20–0.32)	0.13% (0.11–0.16)	0.13% (0.10–0.15)	0.15% (0.12–0.18)
Aortic aneurysm	0.15% (0.14–0.17)	0.20% (0.18–0.21)	0.10% (0.09–0.11)	0.07% (0.07–0.08)	0.10% (0.09–0.11)	0.05% (0.04–0.06)
Other cardiovascular and circulatory diseases	0.14% (0.09–0.17)	0.13% (0.07–0.18)	0.15% (0.08–0.18)	0.07% (0.05–0.08)	0.07% (0.05–0.09)	0.07% (0.05–0.08)
Cardiomyopathy and myocarditis	0.12% (0.09–0.13)	0.12% (0.09–0.15)	0.11% (0.07–0.13)	0.11% (0.08–0.12)	0.11% (0.08–0.13)	0.10% (0.06–0.12)
Endocarditis	0.12% (0.10–0.15)	0.11% (0.09–0.16)	0.14% (0.11–0.18)	0.07% (0.06–0.09)	0.07% (0.06–0.10)	0.08% (0.06–0.10)
Peripheral artery disease	0.01% (0.07–0.03)	0.02% (0.01–0.04)	0.01% (0.01–0.02)	0.02% (0.01–0.03)	0.02% (0.01–0.03)	0.02% (0.01–0.03)

Source: [15] (reproduced under Creative Commons Attribution License [CCBY])

*DALY* disability-adjusted life-year, *UI* uncertainty interval

The onset of infective endocarditis (IE), in general, may imperil the outcome of the structural heart defects (operated or unoperated) claiming a higher than usual mortality and morbidity. The incidence of IE in western population is reported as 1.7–6.2 cases per 100,000 patient years, while the assumed incidence in India based on published studies is expected to be at least 17,000 episodes per year [28].

#### Cardiovascular diseases in adults—coronary artery disease in India

CAD is emerging as the significant cause of morbidity and hampered productivity and substantial mortality amongst the adult population [9]. Prabhakaran et al. analyzed data from three large prospective studies done in India and compared it with GBDS, 2010. They found higher death proportion attributable to CVD (30–42%) and an age-standardized CVD-related death rate (255–525/100,000 [male] and 225–299/100,000 [female]) [14]. Indian data from GBDS (1990–2016) revealed gender-wise percentage of total deaths and DALYs due to CVD and its components (Table 10) [15]. The prevalence of CVD as a whole and ischemic heart diseases times) was 1.3 and 1.6 times higher than the global average, respectively [15]. Figure 3 shows state-wise prevalence of CVD (a), ischemic heart disease (b), stroke (c), and RHD (d). The authors classified states into four ETL groups based on the ratio of DALYs of communicable and non-communicable diseases as follows: high (< 0.3), higher-middle (0.3–0.4), lower-middle (0.41–0.55), lower (0.56–0.75) [15]. The study documented that CVD prevalence was highest in the high ETL state group (Kerala, Punjab, and Tamil Nadu), followed by the higher-middle ETL state group (Andhra Pradesh, Himachal Pradesh, Maharashtra, Goa, and West Bengal) [15]. The study also found a high prevalence of a gamut of risk factors like dietary risks (56,·4%,), high SBP (54,·6%), high total cholesterol (29,·4%), high fasting plasma glucose (16,·7%), and high BMI (14,·7%), as well as tobacco use, less use of fresh fruits, and pollutions. Prevalence of mutation of specific genes (e.g., lipoprotein-related genes, endothelial nitric oxide synthase gene, CYP11B2 and CYP2D6) can also be partly responsible for this epidemiological shift [15, 16].

Adolescent and adult population with CHD (GUCH), Kawasaki disease, and metabolic syndrome have the additional risk of CAD [3, 13, 52, 53]. These cardiac diseases are not covered under the individual health policies [54, 55].

#### Ability vs. disability

Many studies have classified CHD according to their hemodynamic importance [2, 27, 54]. In view of that, the goal of this manuscript is to identify three groups of cardiac patients based on their functional capacity: (1) those with compromised FC; (2) those with normal FC (minor or simple CHD) but get discriminated in fields such as employment, schooling, sports, health and life insurance, due to the mere mention of a diagnosis on their medical records, e.g., patent foramen ovale (PFO), tiny patent ductus arteriosus (PDA), small VSD, and left superior vena cava (LSVC) [2, 27, 54]; and (3) those who achieved normal FC due to natural remission or appropriate treatment.

#### Functional capacity in cardiovascular disorders and cardiac disability

As discussed before, the *c*ardiac ailments are ordained to have changeable waxing-waning functional abilities [1–10, 13]. Therefore, cardiac disability criteria cannot be a single point assessment. Uncorrected heart diseases are usually associated with pressure/volume overload, unbalanced pulmonary and systemic blood flow (Qp/Qs) ratio, cyanosis, PAH, and myocardial ischemia. The ensuing irreversible myocardial damage terminates into intractable arrhythmia, congestive heart failure (CHF) and systemic hypoxia, increasing the risk of any intervention. The combined cardiopulmonary dysfunction in these patients limits the capacity to initiate, sustain, or complete even routine activities. Up to 25% of complex CHDs may present with HF in adulthood [4, 26, 27]. The operated patients may also have suboptimal FC in the presence of residual defects, ventricular dysfunction, chronotropic incompetence, tachyarrhythmia, heart block, SAH, PAH, PVH, prosthesis implantation, post-cardiac transplant deconditioning, IE, and co-existing multi-organ dysfunction [2, 22–25, 28–40]. These patients need to undergo evaluation of cardiac disability based on a scoring system utilizing existing subjective and objective parameters, for the assessment of functional capacity [11–13, 20].

#### Heart disease categories, groups A, B, and C (Tables 1, 5, 6, 7, 8, 9)

Inclusion of cardiac diseases in the HDC Gr A, B, and C is based on hemodynamic significance, type of HD, natural history, amenability to intervention, and overall outcome [2, 3, 5–7, 9, 13, 22–54]. HDC Gr A, B, and C would contribute an entry score of 5, 10, and 20 respectively. In many cases, a detailed diagnosis can foretell the perspective functional capacity. For example, a small PFO would be included in the HDC Gr A (subgroup 1) which suggests no or mild disability (D0 certificate) but PFO with cryptogenic syncope would qualify for HDC Gr B (subgroup 1). The natural history of any HD is predictable to a large extent unless an acquired  factor like bacterial endocarditis superimposes to change the natural history of a HD [28]. The interventions are also important modifier of natural history of a HD, and are expected  to  improve the FC  of a patient  but rarely they may fail to yeild  good results.

#### Subjective criteria—social vs. medical model of disability

Table 2 elaborates on subjective assessment of FC. The disability can be evaluated with the help of subjective scoring of symptoms by age-appropriate methods NYHA for > 14 years of age and modified Ross criteria for < 14 years of age [12, 13, 20, 40–43]. People with a disease are viewed as being disabled by biased behavior of society rather than by their own illnesses [56]. Hence, the patient’s own narration has to be taken into consideration besides the elicitation of clinical signs while evaluating the disability [12, 13, 18, 20]. The fear expressed that a patient may feign symptoms in order to get “disability benefit” must be allayed for the fact that objective criteria are stringent and would prevent any malingering.

#### Objective criteria—elicitation of relevant tests and diligent verification of hospital records

Table 3 shows a systematic approach to assess FC objectively based on existing parameters described in literature [12–14, 44–52]. Examination of longitudinal meticulously kept medical records gives information about the frequency and duration of hospitalization, basal saturation and need for oxygen therapy, polycythemia, documented episodes of syncope and other cardiac events, systemic congestion of cardiac origin, capacity to exercise, episodic elevation of cardiac enzymes and abnormal electrocardiograms, cardiac interventions, need for temporary or permanent pacemaker implantation, co-existing illnesses, and response to medical management. The old echo reports may reveal a pattern of undulating course of cardiac dysfunction and pulmonary hypertension. Echo-imaging also provides surrogate objective parameters like structural and functional abnormalities of the heart, PA pressures, basal or stress-induced regional wall motion abnormality due to reduced coronary perfusion, valvular stenosis or regurgitation, dilatation of ventricles, ventricular dysfunction, pericardial effusion, residual defects, and cardiomyopathy. The information obtained from echo or other imaging modalities is pertinent to diagnosis and are ancillary to the overall assessment of functional capacity. Nevertheless, functional capacity as we know is the ability to initiate and sustain accustomed and unaccustomed exercise, indicating the efficiency of the integrated functioning of the cardiovascular-pulmonary unit, in the presence of a cardiac disease [19–29]. Determination of peak VO2/METs in response to exercise (submaximal treadmill or bicycle exercise testing) or 6-, 9-, and 12-min walk tests are the objective methods described in the literature to assess FC of a patient with the compromised cardiac status [44–50]. High level of N-terminal pro BNP (NT-proBNP) and B-type natriuretic peptide (BNP) levels have been used to grade severity of CHF in the modified Ross classification of HF [42].

#### Unheeded issues of cardiac patients: psychological, social, and financial rehabilitation

An important but often ignored aspect of management of CVD is psychosocial and financial rehabilitation. As discussed before, patients with no hemodynamic issues (HDC group A) may be treated equally with the peer group, in terms of job, insurance, and social acceptance [57, 58]. On the other hand, patients with hemodynamically significant HD (HDC groups B and C) have a myriad of issues related to marriage, pregnancy (women with CHD), education, psychosocial behavior, and employment [58–66]. A German series reported multiple complications in a cohort of 267 pregnant women like high incidence of arrhythmia (12%), deterioration of NYHA class (30%), and premature delivery (12%) [59]. Psychological issues like depression remained undiagnosed in patients with HD [60]. Oh et al. found increased anxiety, depression, and somatization in Korean children with CHD [61]. Malpas et al. reported high judicial encounters (0.9%) in a series consisting of 1640 ACHD (adults with CHD) [62]. Adults (above 25 years of age) with complex CHD were found to have less chance of employment when compared with a peer group (64% vs. 83%) [64]. In a series consisting of 135 adult patients, 88% patients with complex CHD had at least 1 long-term complication (arrhythmia, heart failure, or pulmonary hypertension) [66]. A significant number of patients with complex CHD from this series, had poor NYHA class (38.1%), poor pass rate (45.7%), poor sports activity (50%), and also very low annual salary income (61.5% < 11,500 euros/year) [66]. Consequently, the GUCH population is expected to have a suboptimal social-demographic outcome (educational attainment, employment, and relationship) culminating into psychopathological tendencies (i.e., risk-taking behavior, substance abuse, and other criminal activities) [60–66].

#### Direct and indirect microeconomic effects of a heart disease on affected families leading to debt-entrapment and poverty

Management of cardiac diseases is usually self-financed by the family resulting in catastrophic health spending (CHS).

The microeconomic impact of cardiac surgery on the families of patients with CHD was evaluated; 81% of the families struggled financially and went for distress financing, 4% had health insurance, while 10% were affordable [8]. Even in the post-operative period, 56% of the families needed to change their lifestyle to accommodate the financial burden [8].

#### Rising incidence of cardiovascular diseases

The rising incidence of cardiovascular diseases in adults has an impact on macroeconomic due to loss of the working force owing to morbidity and mortality. Ischemic heart diseases (IHD) and stroke are responsible for > 80% of CVD [14]. India has an estimated age-standardized CVD death rate of 272/100000, higher than the average global mortality [14–17]. Huffman et al. assessed the microeconomic impact of CHS and distress financing of CVD in four countries (India, China, Tanzania, and Argentina) [67]. India had the lowest insurance coverage and highest CHS amongst them [67]. In a nutshell, the microeconomic effects of morbidity cost of CVD include direct (ambulance, hospital, interventions, etc.) and indirect costs (due to loss of working hours and income) [9, 14–17, 67]. Insurance for cardiac diseases has been done successfully in other countries and needs to be replicated in India [12].

#### Need for a multidisciplinary approach for patients going through cardiac rehabilitation programs [3, 4, 11, 12, 26, 27]

A post-intervention, cardiac rehabilitation program improves long-term survival, quality of life, and psychological well-being [68–70]. These programs must have a cost-effective multidisciplinary approach to handle the multiple issues like treatment of co-morbid factors, patient’s (and parent’s) education, physiotherapy, neuro-developmental growth, psychiatric problems, speech, visual and hearing impairment, and underlying genetic syndromes [69, 70].

#### Functional capacity-based sport advisory for patients with heart diseases

Dean et al., in a series of 177 patients (consisted of mild, moderate and severe CHD), found that participation in frequent physical activity and competitive sports led to higher maximum predicted oxygen consumption and relatively lower body mass index (BMI). In this series, 29% of patients with severe HD also participated in competitive sports, defying the Bethesda guidelines. Several such studies from literature now maintain that sports protocols and advisories based on individual’s functional capacity and sport classification may eventually improve quality of life [50, 51].

## Conclusion

In the last few decades, there has been a substantial emphasis on timely diagnosis and appropriate intervention; however, issues related to social security, cardiac rehabilitation, and economic independence have not been given much attention. Social insolence and stigma have been the critical causes of limited opportunities for a patient with heart disease within their family, society, and in the job market. Nonetheless, in an extensive PubMed and Google search, no guidelines were found related to the disability, insurability, and employability of cardiac patients in India. This manuscript is guided by a group of professionals and proposes “CCDS” based on subjective and objective criteria and heart disease category groups. The manuscript uniquely offers a scientific tool to frame the criteria for “disability status” for eligible cardiac patients, to bring cardiac disability in the list of specified diseases under the newly enacted RPWD Act, 2016, in order to empower these patients legally.
